# Adjuvant locoregional radiation therapy in breast cancer patients with pathologic complete response after neoadjuvant chemotherapy: A systematic review and meta-analysis

**DOI:** 10.1016/j.ctro.2021.12.010

**Published:** 2022-01-05

**Authors:** Normehr Nikyar, Eva Tegnelius, Antonis Valachis

**Affiliations:** a Faculty of Medicine and Health, Örebro University, Örebro, Sweden; b Department of Oncology, Faculty of Medicine and Health, Örebro University, SE 70182 Örebro, Sweden

**Keywords:** Breast cancer, Neoadjuvant, Adjuvant radiotherapy, Locoregional radiotherapy, pCR, ypN0

## Abstract

**Purpose:**

The aim of the present meta-analysis was to investigate the impact of adjuvant locoregional radiation therapy (LRRT) in breast cancer patients with clinical lymph node metastatic disease achieving ypN0 after neoadjuvant chemotherapy (NACT).

**Materials and methods:**

A systematic review of studies on PubMed was performed. A meta-analysis was conducted by computing extracted hazard ratios (HRs) and 95% confidence intervals (CIs) into a fixed-effects model.

**Results:**

Thirteen studies were included in the meta-analysis. Adjuvant LRRT significantly reduced the risk of locoregional recurrence (LRR) in patients with N+ at diagnosis and ypN0 (HR 0.59; 95% CI 0.42–0.81). However, no statistically significant difference on disease-free survival (DFS) or overall survival (OS) was found.

**Conclusions:**

LRRT significantly reduced the risk of LRR in patients with ypN0 after NACT whereas no impact on DFS or OS was observed. The low level of evidence should be considered when interpreting the results in clinical practice.

## Highlights

• Adjuvant locoregional radiation therapy (LRRT) is standard of care in pN+ breast cancer.
• Adjuvant LRRT in pN0 after neoadjuvant chemotherapy (NACT) is controversial.
• Thirteen studies were found to be eligible to a meta-analysis.
• Adjuvant LRRT seems to almost halve the risk for locoregional recurrence.
• No improvement in disease-free or overall survival was observed.
• The current level of evidence is low.

## Graphical abstract

**Figure:**
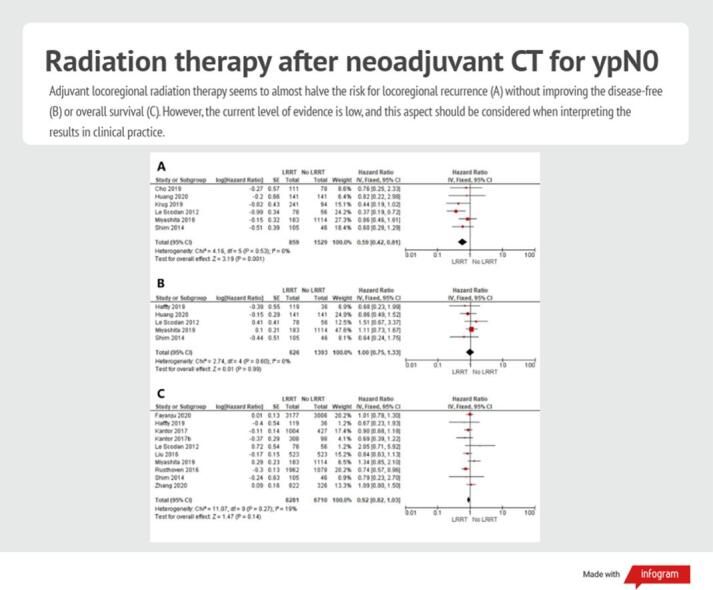

## Introduction

Neoadjuvant chemotherapy (NACT), namely the use of chemotherapy before surgery has been shown to be as effective as adjuvant chemotherapy for patients with operable breast cancer in terms of survival [1]. However, NACT has some potential advantages over adjuvant therapy as the higher probability for breast conserving surgery, and the ability for response-guided treatment both preoperatively and postoperatively [2]. Considering these potential advantages, a significant increase in the use of NACT has been noted over the past ten years [3].

Several randomized trials have shown that patients with large tumors or positive lymph nodes undergoing local or locoregional radiation therapy (LRRT) after adjuvant chemotherapy have a lower risk for locoregional recurrence (LRR) and improved breast cancer survival [4], [5], [6]. However, the benefit of LRRT for breast cancer patients where pathologic complete response (pCR) has been achieved after NACT is not established. According to the National Cancer Institute (NCI), the suitability of LRRT after NACT should be considered for patients with initial N+ breast cancer or lymph node metastatic disease after surgery [7]. However, the prognostic significance of pCR after NACT and the lack of convincing evidence on the impact of LRRT on patients with pCR after NACT has made the usage of LRRT in this setting questionable [8], [9], [10], [11], [12]. In fact, some studies have shown that LRRT in patients with pCR after NACT reduces the risk of LRR, whereas another study have failed to confirm these results [13], [14], [15]. Due to the limited evidence and conflicting results from existing research, there is a lack of consensus regarding the use of LRRT in clinical T3 and/or lymph node metastatic breast cancer patients with favorable response after NACT [16]. In addition, two major sources of bias, namely the confounding by indication and the immortal time bias (ITB), are common in existing studies on this topic and should be considered when interpreting the results.

The aim of this meta-analysis was to gather the current evidence and investigate the impact of adjuvant LRRT on breast cancer patients with clinical T3 and/or lymph node metastatic disease and pCR after NACT.

## Materials and methods

### Literature search and study selection

A systematic review was carried out through a literature search on PubMed until November 2020 limited by studies published in English and after 1990. The following searching algorithm was used: “(radiotherapy OR radiation therapy) AND (neoadjuvant OR preoperative OR induction OR primary) AND (postmastectomy OR postoperative) AND breast cancer”.

To be included in this systematic review and meta-analysis, the studies had to include patients with clinically T3 and/or lymph node metastatic disease at diagnosis, present data on adjuvant radiotherapy in relation to pCR after NACT, and present outcome of adjuvant radiotherapy in terms of LRR and/or disease-free survival (DFS) and/or overall survival (OS). Considering the risk for confounding by indication, namely the risk that adjuvant radiotherapy was selected to patients with more aggressive disease, only studies presenting data from multivariate analyses or propensity score matching were eligible.

The concept of adjuvant radiotherapy in the present study included both the local radiation therapy to the chest wall after mastectomy and the LRRT to breast or chest wall and to regional lymph nodes after mastectomy or breast conserving surgery.

The exclusion criteria were studies that only included patients with inflammatory breast cancer or clinically T4 disease, studies without data on whether adjuvant radiation therapy was given or data on response to chemotherapy and studies without presenting relevant outcomes.

Two independent researchers carried out the literature search and data extraction and consensus through discussion was achieved between the researchers in case of discrepancy.

### Data collection process

The following data were extracted from the studies: first authoŕs surname, year of publication, journal, country, type of study (prospective, retrospective analysis of prospectively collected data, retrospective), multicentric (yes or no); enrollment years, median follow-up, total number of patients, number of patients for adjuvant radiation therapy /no adjuvant radiation therapy; stage at diagnosis, pCR (defined as ypT0 and ypN0), ypN0 defined as pathologic complete response in axilla, breast cancer subtype (triple negative breast cancer (TNBC), HER2-positve, luminal), ITB adjustment (yes or no); hazard ratio (HR) multivariate and 95% confidence interval (CI), or the P-value from matched Kaplan Meier analyses for LRR, DFS, and OS; covariates in multivariate analyses.

### Quality assessment

The included studies were evaluated by using the Risk Of Bias In Non-randomized Studies - of Interventions (ROBINS-I) tool in order to review the limitations, and potential systematic biases of each study [17]. The risk of bias was evaluated within the following eight specific bias domains: Bias due to confounding; bias in selection of participants into the study; bias in classification of intervention; bias due to deviation from intended interventions; bias due to missing data; bias in measurement of outcome; bias in selection of the reported result; overall risk of bias.

Each domain was judged with low, moderate, serious, or critical based upon the risk of bias.

### Certainty of evidence

The Grading of Recommendations Assessment, Development and Evaluation (GRADE) approach was used to assess the evidence of the pooled analyses in the meta-analysis [18]. The certainty of evidence was rated as very low, low, moderate, or high.

### Data synthesis

The meta-analysis was conducted using the Review Manager (RevMan) 5.3 software. Each individual study was weighted using the inverse variance method. Furthermore, logHR and standard error (SE) was calculated. The Tierney method was used to calculate HRs when only P-values from matched Kaplan Meier analyses were present [19]. HR and 95% CI from each individual study were thereafter recalculated. Heterogeneity between the studies was assessed by using Chi^2^ test and I^2^ statistics. Indications for significant heterogeneity were P < 0.05 on the Chi^2^ test, and I^2^ > 50%. The fixed-effects model was selected for calculating the pooled HRs, due to the absence of heterogeneity between the studies. The results of the meta-analysis were graphically presented as forest plots and were considered statistically significant if P < 0.05. Publication bias was evaluated by analyzing asymmetry in SE-based funnel plots.

## Results

### Study selection

**Fig. 1:**
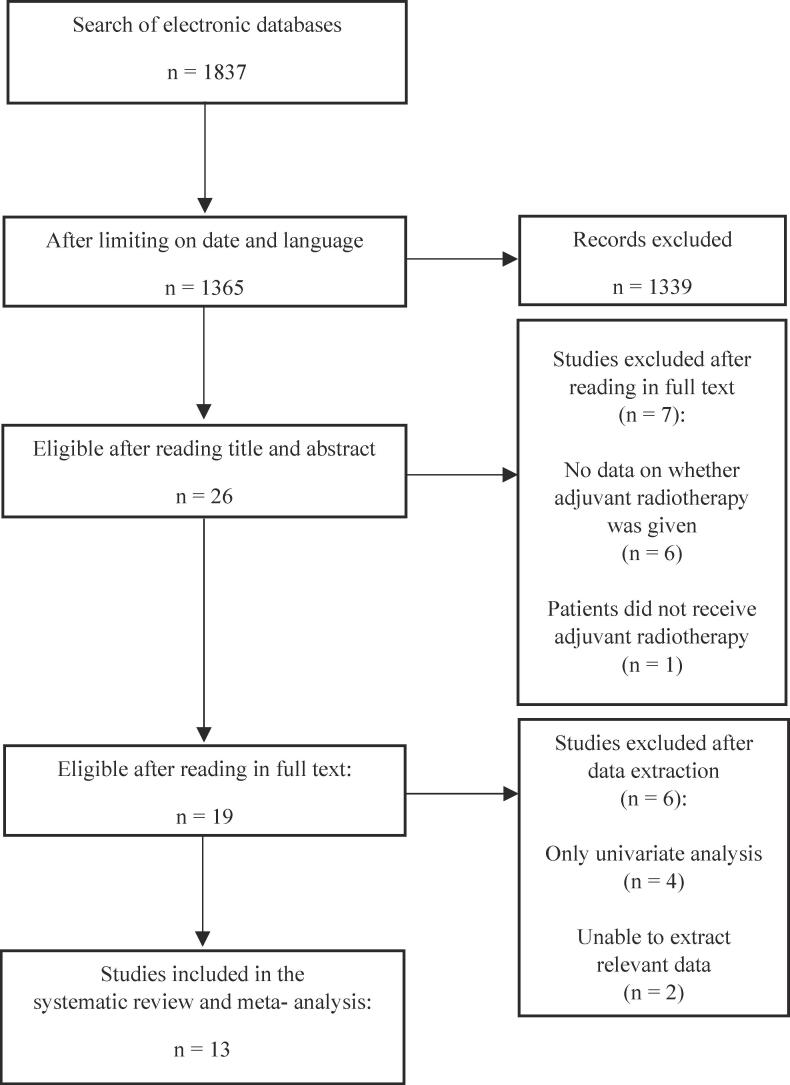
Flowchart diagram of study selection process.

The initial search on PubMed yielded 1837 studies. After applying the inclusion and exclusion criteria, 13 studies were included in the meta-analysis [13], [14], [15], [16], [20], [21], [22], [23], [24], [25], [26], [27], [28]. Fig. 1 summarizes the selection process.

### Study characteristics

Table 1 presents a summary of the characteristics of eligible studies. The median follow-up among the eligible studies ranged between 39 and 91.4 months. All studies were based on breast cancer patients with initial clinical stage T_any_N+ whereas no study specifically addressed the impact of adjuvant radiation therapy after pCR in patients with initially clinical T3N0 breast cancer. The latter research question was an additional aim of our meta-analysis that was unable to be investigated using pooled analyses due to the lack of relevant studies in literature.

**Table 1:** Study characteristics of the 13 eligible studies.

Author (year) [Ref]	Country	Type of study	Multicentric	Enrollment years	Number of patients	Age at diagnosis and molecular subtype in study cohorts	Median follow-up (months)	ITB adjustment	Covariates in multivariate analyses
Cho (2019) [21]	Korea	Retrospective	Yes	2005–2011	189	>50 yrs old 43.4%; Luminal 45.5%, HER2-positive 25.9%, TNBC 28.6%	78.0	No	Grade, LVI, endocrine therapy
Fayanju (2020) [27]	USA	Retrospective with prospectively collected data	Yes	2004–2015	6183	Median age 51 (IQR: 43–60); Luminal 40%, HER2-positive 34.4%, TNBC 24.1%	40.1	No	Age, radiation, race/ethnicity, insurance status, grade, cT stage, Charlson/Deyo comorbidity score, facility type, facility location, extent of axillary surgery, histology, tumor subtype
Haffty (2019) [26]	USA	Retrospective with prospectively collected data	Yes	2009–2011	248	Luminal 59.6%, HER2-positive 32.8%, TNBC 26.3%	70.8	Yes	cT stage, in- breast pCR, tumor biology
Huang (2020) [20]	China	Retrospective	Yes	2000–2014	282	Median age 49 (range; 20–79); Luminal 50.1%, HER2-positive 30.9%, TNBC 19.0%	72.9	No	Age, cT stage, cN stage, LVI, molecular subtype, ypT, endocrine therapy, adjuvant chemotherapy
Kantor (2017) [28]	USA	Retrospective with prospectively collected data	Yes	2004–2008	1937	<50 yrs old 46.1%, 50–70 yrs old 46.6%, >70 yrs old 7.3%	69	No	Age, race, insurance, charlson comorbidity index, histology, grade, ER-status, PR-status, endocrine therapy, cT stage, ypT stage
Krug (2019) [15]	Germany	Retrospective with prospectively collected data	Yes	2002–2010	402	Median age 49 (range: 21–78); Luminal 52.1; HER2-positive 15.3%, TNBC 15.7%	51.5	Yes	cT stage, cN stage, age, ER-status, PR-status, HER2 status, histologic subtype, grading, response to chemotherapy
Le Scodan (2012) [23]	France	Retrospective	No	1990–2004	134	Mean age 49.9 (range: 28–71)	91.4	No	Age, cT stage, cN stage, histologic stage, inflammatory signs, endocrine therapy, NACT regimens, ER-status, PR-status response to NACT
Liu (2016) [16]	USA	Retrospective	No	1998–2009	1046	Median age 50 (range: 20–88)	56.0	Yes	Age, race, insurance status, histologic grade, cT stage, ypT stage, no. of examined regional nodes, clinical stage, endocrine therapy
Miyashita (2019) [24]	Japan	Retrospective with prospectively collected data	Yes	2004–2009	1297	Median age 53 (range: 23–92)	NR	No	Age, cT stage, cN stage, biological subtype
Rusthoven (2016) [25]	USA	Retrospective with prospectively collected data	Yes	2003–2011	3040	Age < 50 yrs old 55.8%, >50 yrs old 44.2%	39	Yes	Age, race, year of diagnosis, Charlson/Deyo comorbidity score, grade, cT stage, in-breast pCR, ypN, extent of axillary surgery, ER-status, endocrine therapy
Shim (2014) [22]	Korea	Retrospective	Yes	1998–2009	151	Median age 47 (range: 27–78); Luminal 41.1%, HER2-positive 13.9%, TNBC 24.5%	59	No	Age, cT stage, cN stage, ypT
Wang (2020) [13]	China	Retrospective	No	2004–2016	48	Median age 50 (range: 23–64); Luminal 54.2%, HER2-positive 19.8%, TNBC 19.8%	72	No	Age, clinical stage
Zhang (2020) [14]	Taiwan	Retrospective with prospectively collected data	No	2007–2015	1423	Median age 51 (IQR: 44–59)	NR	Yes	Age, diagnosis year, Charlson comorbidity index, tumor differentiation, clinical stage, ypT, ypN, NACT regimen, nodal surgery, ER-status, PR-status, HER2-status, hospital type

Abbreviations: Ref, reference number; ITB, immortal time bias; LVI, lymphovascular invasion; cT stage, clinical T stage; pCR pathologic complete response; ypT, pathologic T stage; ER, estrogen-receptor; PR, progesterone receptor; NACT, neoadjuvant chemotherapy; NR not reported; ypN, pathologic N stage; TNBC, triple negative breast cancer; IQR, interquartile range.

There were five studies that adjusted for ITB in their analyses [14], [15], [16], [25], [26]. Two of them used suitable statistical methods to minimize ITB [14], [25], and three adjusted for the bias in their study design [15], [16], [26].

### Risk of bias

The overall risk of bias was judged as serious in 12 out of 13 eligible studies whereas 1 study was judged as moderate overall risk of bias (Table 2).

**Table 2:** Risk of bias within the 13 included studies according to the ROBINS-I tool.

Author (year) [Ref]	Domains of potential bias							
	Confounding	Selection	Intervention classification	Deviation from intervention	Missing data	Measurement of outcome	Selection of reported results	Overall
Cho (2019) [21]	Moderate	Serious	Low	Low	Low	Low	Low	Serious
Fayanju (2020) [27]	Moderate	Serious	Low	Low	Low	Low	Low	Serious
Haffty (2019) [26]	Moderate	Serious	Low	Low	Low	Low	Low	Serious
Huang (2020) [20]	Moderate	Serious	Low	Low	Low	Low	Low	Serious
Kantor (2017) [28]	Moderate	Serious	Low	Low	Low	Low	Low	Serious
Krug (2019) [15]	Moderate	Serious	Low	Low	Low	Low	Low	Serious
Le Scodan (2012) [23]	Moderate	Serious	Low	Low	Low	Low	Low	Serious
Liu (2016) [16]	Moderate	Moderate	Low	Low	Low	Low	Low	Moderate
Miyashita (2019) [24]	Moderate	Serious	Low	Low	Low	Low	Low	Serious
Rusthoven (2016) [25]	Moderate	Serious	Low	Low	Low	Low	Low	Serious
Shim (2014) [22]	Moderate	Serious	Low	Low	Low	Low	Low	Serious
Wang (2020) [13]	Moderate	Serious	Low	Low	Low	Low	Low	Serious
Zhang (2020) [14]	Moderate	Serious	Low	Low	Low	Low	Low	Serious

Abbreviations: Ref, reference number.

### Impact of LRRT on LRR, DFS, and OS in patients with axillary pCR

**Fig. 2:**
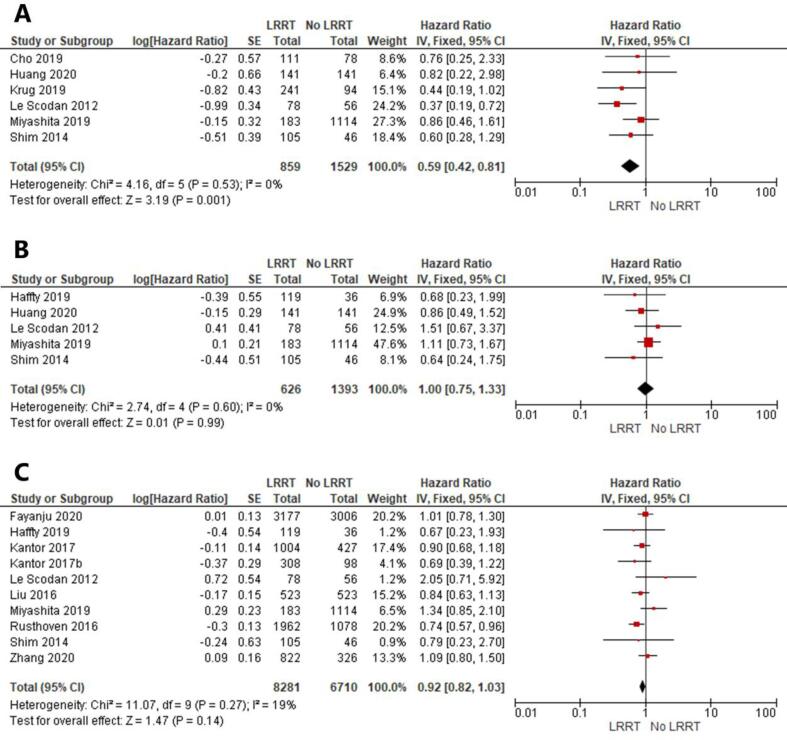
Forest plots on the impact of locoregional radiation therapy (LRRT) on A. locoregional recurrence, B. disease-free survival, C. overall survival, in patients with ypN0 after neoadjuvant chemotherapy. In C, Kantor et al. presented separate data on cN1 and cN2 breast cancer without overlapping between the two cohorts and was included as two separate studies (Kantor 2017 for cN1; Kantor 2017b for cN2).

**Fig. 3:**
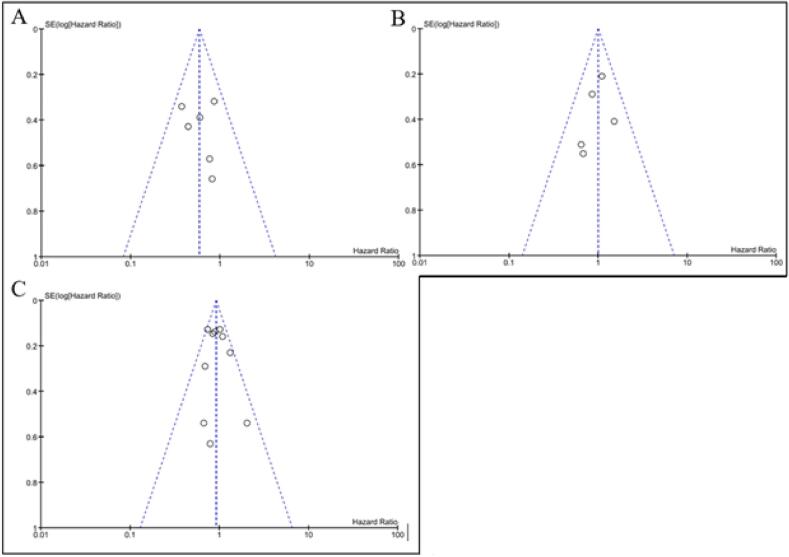
Funnel plots regarding A. 6 studies that reported the impact of locoregional radiation therapy (LRRT) on locoregional recurrence, B. 5 studies that reported the impact of LRRT on disease-free survival, C. 9 studies that reported the impact of LRRT on overall survival, in patients with ypN0 after neoadjuvant chemotherapy.

The analysis on the impact of LRRT on LRR included six studies (Fig. 2A) [15], [20], [21], [22], [23], [24]. In total, 2388 patients with N+ at diagnosis and ypN0 after NACT were included in the analysis, 859 received LRRT and 1529 did not. The results showed a statistically significant reduced risk of LRR in patients who received LRRT (HR 0.59; 95% CI 0.42–0.81; P = 0.001). The symmetric funnel plot indicated that no publication bias was present (Fig. 3A). The certainty of evidence in the pooled analysis was evaluated as low by GRADE (Table 3).

**Table 3:** Certainty of the evidence on pooled analyses according to GRADE-approach.

Outcomes	Relative effect (95% CI)	No. of participants (studies)	Certainty of the evidence (GRADE)
Locoregional recurrence	HR 0.59 (0.42 to 0.81)	2388 (6 observational studies)	⊕⊕ LOW
Disease-free survival	HR 1.00 (0.75 to 1.33)	2019 (5 observational studies)	⊕ VERY LOW
Overall survival	HR 0.92 (0.82 to 1.03)	14,991 (9 observational studies)	⊕ VERY LOW

Abbreviations: CI, confidence interval; HR, hazard ratio; No., number.

Five studies investigated the role of LRRT on DFS were eligible (Fig. 2B) [20], [22], [23], [24], [26]. A total of 2019 patients were included in the analysis out of which 626 received LRRT and 1393 did not. The results showed no statistically significant difference between the LRRT and no LRRT groups (HR 1.00; 95% CI 0.75–1.33; P = 0.99). The symmetric funnel plot indicated that no publication bias was present (Fig. 3B). The certainty of evidence in the results from this analysis was evaluated as very low by GRADE (Table 3).

Regarding the impact of LRRT on OS, nine studies were eligible including 14,991 patients out of which 8281 were treated with LRRT and 6710 without LRRT (Fig. 2C) [14], [16], [22], [23], [24], [25], [26], [27], [28]. The pooled HR showed no statistically significant difference between the LRRT and no LRRT groups (HR 0.92; 95% CI 0.82–1.03; P = 0.14). The symmetric funnel plot indicated that no publication bias was present (Fig. 3C). The certainty of evidence in the results from this analysis was evaluated as very low by GRADE (Table 3).

In all three meta-analyses regarding the impact of LRRT on LRR, DFS, and OS, respectively, we performed sensitivity analyses by excluding one study where patients with cN0 were also included in the study cohort [23], and found very similar results (data not shown).

### Subgroup analyses

A subgroup analysis was performed including three studies with data on the impact of LRRT on LRR in patients with pCR (both ypT0 and ypN0) [13], [14], [15]. In total, 390 patients were included in the analysis, 242 received LRRT and 148 did not. The results showed a statistically significant lower risk of LRR in patients who received LRRT (HR 0.24; 95% CI 0.11–0.49; P < 0.0001).

Considering the risk of ITB in studies investigating adjuvant radiotherapy, an additional subgroup analysis was performed including only studies that presented results on OS where ITB was mitigated by study design or suitable statistical analysis. In total, four studies were eligible for this subgroup analysis including 5389 patients, 3426 received LRRT and 1963 did not [14], [16], [25], [26]. The results showed no statistically significant difference between the LRRT and no LRRT groups in terms of OS (HR 0.86; 95% CI 0.73–1.01; P = 0.06).

## Discussion

This meta-analysis showed a statistically significant reduced risk of LRR when receiving LRRT in breast cancer patients with N+ at diagnosis and ypN0 after NACT whereas no survival benefit was observed regarding DFS or OS. However, the certainty of evidence from the pooled analyses ranges from very low to low, thus jeopardizing the implication of the results into daily clinical practice.

The study design of the eligible studies is prone to two major risks of bias that could influence the results, namely confounding by indication and ITB. To mitigate the risk of confounding by indication in our meta-analysis, we included only primary results from multivariate analyses or results after adequate matching method. Although the risk for unmeasured confounding in observational studies remains despite the adequate use of statistical methods, the pooled analyses of multivariate results provide a more reliable approach.

ITB can cause an inaccurate survival benefit among patients that receive adjuvant radiotherapy, because patients must survive until the start of the intervention in order to be included in the adjuvant radiotherapy group, meanwhile patients that die before the start of the intervention are included in the no adjuvant radiotherapy group [29]. To investigate how ITB could impact our meta-analysis, we performed a subgroup analysis including only studies that used methods to mitigate the risk of ITB. Considering the comparable pooled OS results between the main analysis and the subgroup analysis with only studies adjusted for ITB we can hypothesize that ITB does not seem to represent a major drawback in our meta-analysis.

Recently, a meta-analysis by the Italian Association of Radiation therapy and Clinical Oncology investigating the same research question was published [30]. The authors could not find any difference in LRR, DFS, or OS when adjuvant radiation therapy was added in patients with pCR after NACT. However, the later meta-analysis did not consider the potential impact of confounding by indication or immortal-time bias on the primary analyses of the eligible studies and, as a result, the pooled analyses have a high level of uncertainty.

The lack of survival benefit with the addition of LRRT in patients with ypN0 despite the potential benefit in LRR observed in the present meta-analysis is somewhat unexpected and challenging to explain. The inclusion of different study cohorts in the pooled analyses for LRR and OS respectively based on the available results within each study as well as the lack of adequate follow-up to observe survival differences might be some potential explanations. However, an inherent risk of bias in pooled analyses due to the low certainty of evidence, as it clearly observed using the GRADE approach, might also be the driving factor for this discrepancy.

All analyses in this study were based on breast cancer patients with initial lymph node metastatic disease achieving ypN0 and therefore, the impact of adjuvant radiation therapy in breast cancer patients with initial T3N0 breast cancer that achieve pCR after NACT remains unclear. However, in a systematic review of 24 studies, 23 of which were single-institution retrospective cohorts, it was suggested that omitting postmastectomy radiation therapy (PMRT) can be considered in patients with T3N0 breast cancer with pCR in breast and axilla after NACT. The systematic review proposed some groups of breast cancer patients who were at low risk of LRR if PMRT was omitted after NACT and breast cancer patients with T3N0 at diagnosis with pCR after NACT were considered at low risk of LRR [12]. However, this systematic review did not consider confounding by indication bias or ITB when selecting the eligible studies and, as a result, there is a high level of uncontrollable uncertainty in the summary of the evidence.

Following the efforts to more personalized treatment approach rather than the “one-size fits all” approach in cancer therapy, one could argue that there should be subgroups of patients achieving ypN0 after NACT that will not benefit at all from LRRT whereas others will be benefited. In our meta-analysis, we were able to investigate whether patients with pCR in both breast and axilla could represent a group without any benefit from LRRT. However, we observed a benefit with LRRT in terms of LRR that was similar with the benefit on LRR observed in our primary analysis of patients achieving ypN0.

We were unable to conduct further subgroup analyses based on different patient- or tumor characteristics due to the lack of adequate data from primary studies. However, a retrospective study from the National Cancer Data Base (NCDB) including 8321 patients with lymph node metastatic breast cancer (cN1-2 stage) showed that LRRT improved OS in patients with hormone-receptor negative disease, and ypN0 after NACT (HR 0.65; 95% CI 0.48–0.88; P < 0.01) whereas no OS benefit was observed in the whole study cohort including patients with different breast cancer subtypes [28].

Two additional limitations of the present meta-analysis that deserve attention is the fact that our pooled analyses were based on study-level results and not on patient-level data that would give the possibility to investigate the research question within different patient subgroups. Furthermore, the lack of relevant information regarding irradiated target volumes within primary studies is a source of clinical heterogeneity among eligible studies that could potentially influence the results.

Considering the high risk of bias of the eligible studies, especially the risk for confounding by indication and ITB, it was expected that the certainty of evidence from the present meta-analysis ranges from very low to low. The most suitable way to overcome these biases and provide high level of evidence is to perform well-designed randomized trials. In fact, the ongoing NSABP B-51/RTOG 1304 randomized trial will be able to answer to the research question on the role of adjuvant radiotherapy after NACT and pCR using randomized evidence [31].

So, how does one interpret the results of the present meta-analysis while anticipating for the results of the ongoing randomized trial? Using well-defined approaches to grade the level of evidence, this meta-analysis provides the current evidence on this research question from studies that tried to mitigate the risk of major biases on their analyses. Our results accompanied with their level of evidence can be used by the clinicians in the discussion with the breast cancer patients for a shared decision making on this complex clinical situation until the evidence from the randomized trial is available.

This meta-analysis including studies that tried to mitigate the risk for confounding by indication bias and ITB showed that receiving LRRT significantly reduced the risk of LRR in patients that achieve ypN0, and pCR in both breast and axilla after NACT whereas no impact was found on DFS or OS. However, the results should be interpreted in clinical practice with caution considering the low certainty of evidence. Results from the ongoing randomized trial are anticipating to provide results with high level of evidence for this complex clinical situation.

## Declaration of Competing Interest

The authors declare that they have no known competing financial interests or personal relationships that could have appeared to influence the work reported in this paper.
